# Biocomputational prediction of non-coding RNAs in model cyanobacteria

**DOI:** 10.1186/1471-2164-10-123

**Published:** 2009-03-23

**Authors:** Björn Voß, Jens Georg, Verena Schön, Susanne Ude, Wolfgang R Hess

**Affiliations:** 1 University of Freiburg, Faculty of Biology, Genetics and Experimental Bioinformatics, Schänzlestr. 1, D-79104 Freiburg, Germany; 2 Freiburg Initiative in Systems Biology, Schänzlestr. 1, D-79104 Freiburg, Germany

## Abstract

**Background:**

In bacteria, non-coding RNAs (ncRNA) are crucial regulators of gene expression, controlling various stress responses, virulence, and motility. Previous work revealed a relatively high number of ncRNAs in some marine cyanobacteria. However, for efficient genetic and biochemical analysis it would be desirable to identify a set of ncRNA candidate genes in model cyanobacteria that are easy to manipulate and for which extended mutant, transcriptomic and proteomic data sets are available.

**Results:**

Here we have used comparative genome analysis for the biocomputational prediction of ncRNA genes and other sequence/structure-conserved elements in intergenic regions of the three unicellular model cyanobacteria *Synechocystis *PCC6803, *Synechococcus elongatus *PCC6301 and *Thermosynechococcus elongatus *BP1 plus the toxic *Microcystis aeruginosa *NIES843. The unfiltered numbers of predicted elements in these strains is 383, 168, 168, and 809, respectively, combined into 443 sequence clusters, whereas the numbers of individual elements with high support are 94, 56, 64, and 406, respectively. Removing also transposon-associated repeats, finally 78, 53, 42 and 168 sequences, respectively, are left belonging to 109 different clusters in the data set. Experimental analysis of selected ncRNA candidates in *Synechocystis *PCC6803 validated new ncRNAs originating from the *fabF-hoxH *and *apcC-prmA *intergenic spacers and three highly expressed ncRNAs belonging to the Yfr2 family of ncRNAs. Yfr2a promoter-*luxAB *fusions confirmed a very strong activity of this promoter and indicated a stimulation of expression if the cultures were exposed to elevated light intensities.

**Conclusion:**

Comparison to entries in Rfam and experimental testing of selected ncRNA candidates in *Synechocystis *PCC6803 indicate a high reliability of the current prediction, despite some contamination by the high number of repetitive sequences in some of these species. In particular, we identified in the four species altogether 8 new ncRNA homologs belonging to the Yfr2 family of ncRNAs. Modelling of RNA secondary structures indicated two conserved single-stranded sequence motifs that might be involved in RNA-protein interactions or in the recognition of target RNAs. Since our analysis has been restricted to find ncRNA candidates with a reasonable high degree of conservation among these four cyanobacteria, there might be many more, requiring direct experimental approaches for their identification.

## Background

In bacteria, non-coding RNAs (ncRNAs) are a heterogeneous group of sequence-specific regulators of gene expression, normally lacking a protein-coding function. They are typically 50–250 nucleotides in length [1], and regulate mRNA translation or decay but sometimes also directly modulate certain protein functions. Most stress responses in the organism best-studied in this respect, *E. coli*, include at least one small regulatory RNA as part of the regulon [2]. However, their functions also include the control of plasmid and viral replication [3], bacterial virulence [4], quorum sensing [5], or the acquired resistance against bacteriophages [6].

In many cases, these ncRNAs function through sequence-specific base pairing; hence they frequently have a (partial) base complementarity to their target RNA molecules. The vast majority of known ncRNAs is encoded at genomic locations far away from their target genes. However, some ncRNAs are transcribed from the reverse complementary strand of the respective target and hence these are fully or partially overlapping with their target RNAs, constituting the class of antisense RNAs. Except for the more common types of ncRNA (ribosomal RNA, tRNA, tmRNA, 6S RNA, RNAse P RNA and *ffs *RNA), genes encoding ncRNAs are not annotated during standard genome analysis. The efforts to accomplish their identification in bacteria can broadly be divided into (i) sequencing the population of small RNAs or (ii) prediction by bioinformatics tools (mostly) followed by experimental verification (see [7] for review). As a result of such systematic searches, more than 80 ncRNAs are now known in *E. coli*, most of which had been overlooked by traditional genome analysis.

Cyanobacteria currently raise considerable interest as they perform oxygenic photosynthesis, fix atmospheric CO_2 _and nitrogen, frequently produce large quantities of bioactive secondary metabolites and due to their potential for the production of biofuels. As long as there is sufficient light available for photosynthesis, cyanobacteria populate widely diverse environments such as freshwater, the oceans, rock surfaces, desert soil or the polar regions. Their adaptation to vastly different environmental conditions suggests the existence of sophisticated regulatory mechanisms. Therefore, various types of regulatory RNA can be expected that interplay with the different signal transduction pathways and stress responses. Indeed, computational-experimental screens based on comparative genome analysis identified seven different ncRNAs in the marine cyanobacteria *Prochlorococcus *and *Synechococcus *[8] which were called Yfr1-7 for c**Y**anobacterial **F**unctional **R**NA. In a follow-up study making use of high density microarrays and exploiting the genome information from meanwhile 12 different *Prochlorococcus *genome sequences, additional 14 ncRNAs and 24 antisense RNAs were found [9]. Unicellular marine cyanobacteria of these genera provide an excellent dataset for computational predictions that require comparative genome information since currently 22 different genome sequences from very closely related isolates are available [10,11]. However, a major bottleneck in the work with these marine cyanobacteria is that despite some recent progress [12], protocols for genetic manipulation are very slow or not available at all. Therefore, the finding that two of these ncRNAs are phylogenetically widely distributed enabled direct genetic work on their functional relevance: Yfr1 is distributed throughout the cyanobacterial radiation [13] and might play a role in the adaptation to redox stress or the regulation of carbon uptake [14], whereas Yfr7 was identified as the homolog of the 6S RNA [15] which is found in all eubacteria [16]. However, for efficient genetic and biochemical analysis of cyanobacterial ncRNAs it would be very desirable to identify a set of ncRNA candidate genes in model cyanobacteria that are easy to manipulate and for which extended mutant, transcriptomic and proteomic data sets are available. In addition to Yfr1 which exists in all four unicellular cyanobacteria targeted here [13], the only currently known ncRNAs in model cyanobacteria are an antisense RNA covering the ferric uptake regulator gene *furA *in *Anabaena *PCC 7120 over its full length [17], and the antisense RNA IsrR, regulating the gene for the light-absorbing protein IsiA under conditions of iron limitation and redox stress in the unicellular *Synechocystis *PCC 6803 [18].

In recent years, comparative genomics-based prediction of ncRNA genes has become a standard method to search for such genes within bacterial genomes [8,19-23]. Thus, the availability of genome sequences from closely enough related species is a critical factor as is the conservation of ncRNAs. In case of unicellular cyanobacterial model organisms, the lack of genome sequences from close relatives has been hampering such studies. With the recent release of the *Microcystis aeruginosa *NIES-843 genome [24], however, a cyanobacterium relatively close to *Synechocystis *has been sequenced.

Here we set out to identify possible ncRNA genes and other RNA elements (5' leader sequences and riboswitches) in the three unicellular model cyanobacteria *Synechocystis *PCC6803, *Synechococcus elongatus *PCC6301 and *Thermosynechococcus elongatus *BP1 plus the toxic *Microcystis aeruginosa *NIES-843 (from now on: *Synechocystis, Synechococcus, Thermosynechococcus *and *Microcystis*) by biocomputational comparative genome analysis with a focus on *Synechocystis*.

## Results and Discussion

### Computational screening for novel ncRNAs

**Figure 1:**
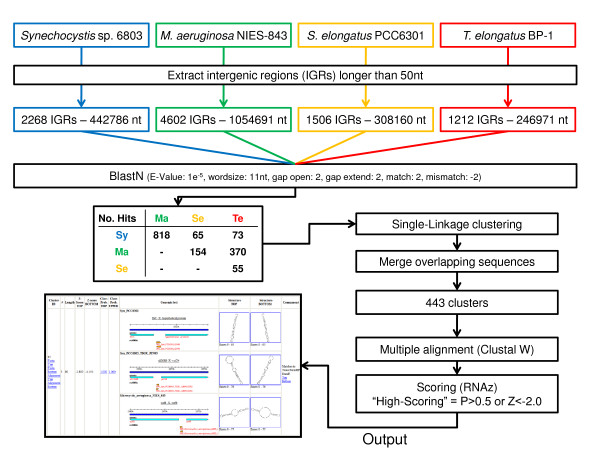
**Pipeline for comparative prediction of RNA elements and ncRNAs**. Intergenic sequences of at least 50 nt were gathered from four cyanobacterial genomes and locally aligned using BLASTN. Sequences which directly produced a significant blast hit (E-value < 10^-5^) or which were connected by a chain of such hits were gathered into clusters ("single-linkage clustering"). After an additional unification step of overlapping sequences within each cluster the resulting clusters and their complement clusters were scored using RNAz [26]. The inset shows how this information is provided in the internet, together with the location within the compared genomes, using the top-scoring cluster (CLID 80) as an example.

To screen for novel RNA elements, all intergenic regions >50 nt were extracted from the four genomes and analyzed as outlined in Fig. 1, leaving out all annotated genes, including tRNA and four structural RNA genes. Although single sequence elements and families of sequence elements that are specific to a single genome cannot be found by our approach, this procedure initially returned 443 predicted clusters holding 1528 individual sequences, from which 383 belonged to *Synechocystis*, 168 to *Synechococcus*, 168 to *Thermosynechococcus *and 809 to *Microcystis*. However, these numbers were diminished in further filtering steps (see below).

The analysis was basically focused on sequence and structure similarities. Detailed information on all clusters predicted by our method including the positions of all sequences is available online [25]. This information, which we show exemplarily in the inset in Fig. 1, includes the location within the compared genomes, flanking genes, a secondary structure prediction as well as Z-scores and probabilities in either forward (Z, P) or reverse (Z rev, P rev) orientation as computed by RNAz [26]. Furthermore we conducted searches against Rfam [27], the database collecting ncRNAs, and TransTermHP [28], of which the results are also given in the online material.

### High-scoring putative RNA elements

**Figure 2:**
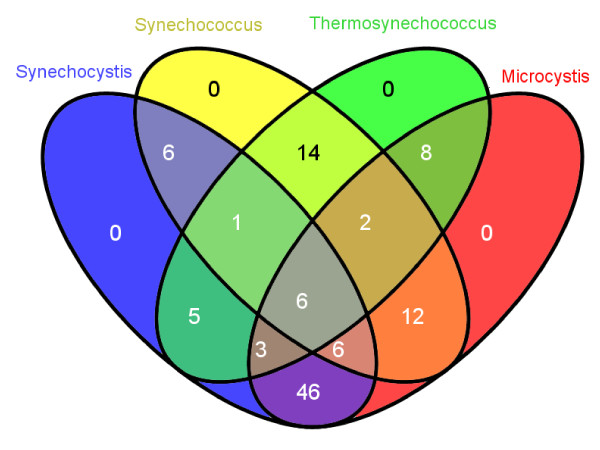
**Venn diagram showing the numbers of predicted sequence clusters that receive high support (P > 0.5 and/or Z score < -2.0) and their distribution along the different genome combinations**. The maximum number of 46 elements is shared between *Synechocystis *and *Microcystis *and only six are present in all genomes. One of these is Yfr1, other examples are CIRCE (Fig. 3) and the Yfr2 ncRNA family (Fig. 5). The figure was produced using Venny [32].

Filtering with P > = 0.5 or Z < = -2.0 reduced the initial number of 1528 individual sequences in the 443 predicted clusters to 113 sequence clusters with 620 individual elements, 94, 56, 64 and 406 in *Synechocystis*, *Synechococcus*, *Thermosynechococcus *and *Microcystis*, respectively. A summary of the highest scoring clusters is given in Table 1[29-31]. The Venn diagram in Fig. 2[32] shows that more homologs were detected in the *Synechocystis*/*Microcystis *comparison than in any other pairwise combination, reflecting the phylogenetic relationships between these species pairs.

**Table 1:** List of selected top-scoring RNA elements (see complete list at [25]).

**CLID**	**Seq #**	**Species**				**(nt)**	**Z**	**Zrev**	**P**	**Prev**	**Comment/Location**
								
		**6803**	**6301**	**BP1**	**NIES**						
80	3	1	1	0	1	80	-2.8	-4.2	1.0	1.0	***sll0088-x-ycf24*, Fig. 3A**

3	2	1	0	0	1	135	-1.1	-3.4	0.992	1.0	*slr0374-x-slr0376*

129	2	0	1	1	0	84	-3.2	-2.2	1.0	0.667	*dnaN-x-cbbZp *(6301)

146	2	1	0	1	0	59	-2.9	-3.5	1.0	1.0	*fus-x-slr1464*

216	2	1	0	0	1	211	-3.8	-2.1	1.0	1.0	Rfam: Conserved RNA structure [29], 5'*speB*

196	2	0	1	0	1	148	-3.5	-2.8	1.0	1.0	*trxA-x-hisA *(6301)

311	2	0	1	1	0	66	-2.8	-2.9	1.0	1.0	*tll0447-x-tll0448 *(BP1)

207	2	0	1	1	0	152	-2.6	-1.5	1.0	0.989	*rpl1-x-rpl10*, *rpl10 *leader [31]

96	4	2	0	0	2	42	-3.9	-3.2	1.0	1.0	possible Rho-indp. term.

56	2	1	0	0	1	52	-2.4	-2.4	1.0	1.0	*slr1739-x-slr1740*

169	3	1	0	0	2	55	-2.3	-2.6	0.026	1.0	possible Rho-indp. term.

139	4	1	1	1	1	242	-1.4	-4.3	0.95	1.0	**Yfr1 **[13]**, 5' *trxA *in 3/4**

338	2	0	1	0	1	69	-2.6	-3.7	1.0	0.994	*syc1122_c-x-cdsA *(6301)

37	3	1	0	0	2	82	-4.7	-4.5	1.0	1.0	*sll0834-x-sll0833*

117	2	1	0	0	1	61	-2.0	-1.4	1.0	0.963	*groEL-2-x-sll0415*

294	2	1	0	1	0	54	-2.6	-1.0	1.0	0.999	**SyR2, Fig. 4B**

86	2	0	1	1	0	42	-2.0	-2.0	1.0	0.999	*nrtA-x-nrtB *(6301)

149	4	2	0	1	1	147	-2.9	-0.9	1.0	0.013	Rfam: thiamin riboswitch [30], 5' *thiC*

394	2	1	0	0	1	110	-2.8	-1.9	1.0	0.996	possible *rps2 *5'UTR

192	2	1	1	0	0	31	-2.8	-3.9	0.996	1.0	possible Rho-indp. term.

107	2	1	0	0	1	224	-1.7	-2.1	0.999	1.0	Rfam: possible riboswitch [29]

302	2	1	0	0	1	96	-4.2	-5.2	1.0	0.995	*slr0708-x-sll0668*

426	2	0	0	1	1	140	-1.8	-2.2	0.997	1.0	*tlr0843-x-plsX *(BP1)

375	2	1	0	0	1	54	-0.3	-0.6	0.999	0.004	*rpl11-x-rpl1*

62	5	1	2	1	1	124	-1.5	-2.3	0.999	0.801	***groES*mRNA leader (CIRCE element) in 4/5, Fig. 3B**

**8 intermediate clusters!**											

219	8	3	2	1	2	200	-2.0	-3.2	0.983	0.997	**Yfr2 family, Fig. 5**

**17 intermediate clusters!**											

159	4	1	1	0	2	186	-2.0	-1.4	0.933	0.411	**SyR1, Fig. 4A**

The total number of sequences in each cluster and the distribution within the four compared genomes plus the total alignment length (nt) is given. The elements are ordered according to the highest RNAz [26] probability in either forward (P) or reverse (P rev) orientation. The closer the probability to 1.0, the more support for structural conservation as an RNA element. Note: hits restricted to *Microcystis *or repeat elements bordering transposable elements have not been included. Location is given for *Synechocystis *PCC 6803 if not indicated otherwise.

We previously showed the existence of Yfr1 in three out of four tested marine cyanobacteria belonging to the genera *Prochlorococcus *and *Synechococcus *[8] and later demonstrated its existence throughout the cyanobacterial radiation, including the four unicellular cyanobacteria targeted here [13]. It was, therefore, no surprise to find Yfr1 among the top-scoring elements (Z-score and probability of -4.340 and 1.0) in cluster 139 (Table 1). Although RNA elements in cyanobacteria are only scarcely covered by Rfam, the existing entries provided another positive control set: the thiamine riboswitch was correctly identified in three strains (cluster 149; Table 1) and also two RNA elements of unknown function were correctly found for *Synechocystis *and *Microcystis *but not for the other two cyanobacteria (cluster 216 and 107 in Table 1). However, we noted the functional role assumed for one of these conserved RNA structures, the *ykkC/yxkD *element (cluster 216), to switch efflux pumps and detoxification systems in response to harmful environmental molecules, may not apply to the cyanobacterial homologs since they are neither in *Synechocystis *nor in *Microcystis *located upstream of a putative transporter gene.

### Synteny among high-scoring RNA elements

**Figure 3:**
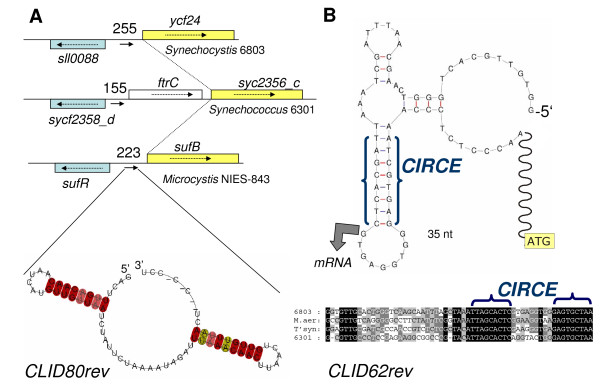
**Types of predicted elements**. **A**. The genomic location of predicted RNA element in cluster 80 and synteny around this element is shown. This element is slightly more likely to be transcribed from the forward strand as indicated by the direction of the arrow within the IGR. The length of the intergenic spacer is given in nt and homologous genes are colour-coded. In *Synechococcus *6301 an *ftrC *gene has been inserted into this region relative to the other. The predicted consensus structure of the RNA element (bottom) consists of two stem-loops separated by a 17 nt single-stranded region. The degree of sequence conservation is colour-coded. **B**. Four of the five sequences in cluster 62 are located upstream of the *groES *operon. This region is known to contain the palindromic CIRCE element and indeed, this element constitutes a critical part of the conserved sequence and structure. The initiation site of transcription of the *groES *mRNA was mapped by 5' RACE to occur from the first G within the nine nt loop that is part of the CIRCE element (bold arrow). The fifth sequence has no CIRCE element but has been clustered into cluster 62 based on other sequence features. At the bottom right, the perfect conservation of the CIRCE element in the four compared cyanobacteria is shown.

The genomic location of a predicted ncRNA gene or RNA element in the same sequence neighbourhood in some or all of the studied cyanobacteria can also be a powerful tool for finding related ncRNAs. Among the 25 high-scoring sequence clusters in Table 1, 9 (36%) showed at least partial synteny. The high scoring element in cluster 80 illustrates this fact. The primary annotation gives no hint about the possible relatedness of the flanking genes. The flanking gene *sufR *annotated in *Microcystis *encodes an iron-sulfur cluster biosynthesis transcriptional regulator and similarity searches revealed that sll0088, syc2358d and *sufR *actually are orthologs of each other (Fig. 3A). Flanking the intergenic region with the predicted RNA element on the other side, genes *ycf24 *and *sufB *are clearly homologs of each other, whereas *ftrC *in *Synechococcus *is not. Yet, *ftrC *has been inserted in this genomic region as the proximate gene, syc2356_c, codes for the homolog of *sufB *and *ycf24*. Thus, the synteny among neighbouring genes clearly support the element predicted in cluster 80 as an orthologous RNA element between the three species. Other cases of partial synteny in flanking genes are observed in cluster 139 since *trxA *is present in 3 out of 4 cases and in cluster 216 with the orthologs *speB *(*Synechocystis*) and agmatinase (*Microcystis*), whereas all other genes are different. Special cases of synteny are exposed in cluster 207 (*rpl10 *leader), 149 (thiamine riboswitch upstream of *thiC*), 394 (*rps2 *leader) and 62 (upstream *groES*). These four examples represent structurally conserved sequence elements upstream of a protein-coding gene to whom they are functionally connected; among them one riboswitch and two ribosomal leaders, thus this position must be conserved. The fourth example, the element upstream of *groES *contains the palindromic CIRCE element (Fig. 3B) thought to bind the heat-shock repressor protein HrcA [33]. Here, we mapped the *groES *transcriptional start site to the first nt of the nine nt loop predicted by secondary structure analysis (Fig. 3B), confirming the previously determined start site [34]. These examples illustrate the variety of elements that become identified by our approach.

### Experimental verification

**Figure 4:**
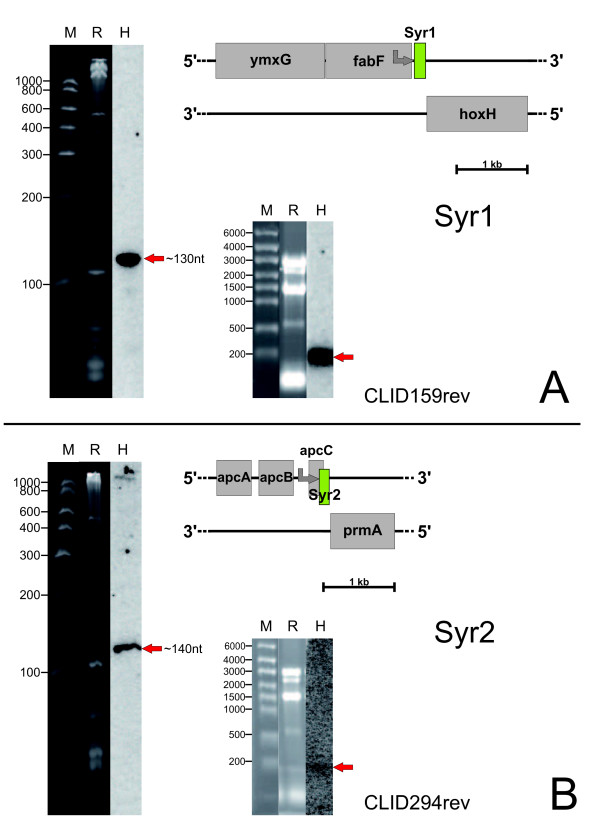
**Experimental verification of two differently scoring ncRNAs by Northern hybridization**. **A**. The element predicted in cluster 159 is transcribed from the forward strand (green), in the same direction as the preceding *fabF *(slr1332) gene. Six 5'RACE sequences support the transcript start of SyR1 to be located 55 nt 3' of the *fabF *reading frame at position 1671919 (grey arrow). Two different blots are shown, one in which RNA was separated in an high-resolution polyacrylamide gel and one resulting from an agarose gel. **B**. The element predicted with the CLID 294 was named SyR2. This ncRNA is longer than the IGR where it is encoded (~140 nt versus 94 nt spacer length). A transcript start was found by 5' RACE within *apcC*, 49 nt before the end of *apcC *(grey arrow). The schemes are drawn to scale. All protein-coding genes are displayed in gray, all ncRNA genes in green; M, molecular mass marker, R, lane in the RNA gel before blotting, H, hybridization.

For exemplary experimental verification of predicted ncRNA genes we chose two very different examples, one well-supported candidate with three members from cluster 159 (probability 0.933 and Z-score -2.00; Table 1) and one from cluster 294 (probability 1.0 and Z-score -2.64; Table 1). Northern hybridization of total RNA from *Synechocystis *using strand-specific RNA probes confirmed the existence of both ncRNAs (Fig. 4). Since we verified the existence of both ncRNAs experimentally, we decided to name these two ncRNAs SyR1 and SyR2, for ***Sy****nechocystis *nc**R**NA **1 **and **2**. SyR1 is a strongly accumulating ncRNA transcribed from a gene in the *fabF *– *hoxH *IGR in the forward direction as the preceding gene *fabF *(Fig. 4A). The *syr1 *gene corresponds with a length of ~130 nt to about two thirds of the *fabF-hoxH *intergenic spacer (length 206 nt). Judged by Northern hybridization, there was no evidence for a possible cotranscription with *fabF*. The element predicted with the CLID 294 is located 3' to a protein-coding gene, too, and is transcribed from the forward strand in *Synechocystis *6803. SyR2 is an ~140 nt ncRNA transcribed from a gene in the *apcC *(ssr3383) – *prmA *(sll1909) IGR in the same forward direction as the preceding gene *apcC*. SyR2 is accumulated to rather high amounts, too, but these appeared lower than in case of SyR1 (Fig. 4B). The preceding *apcC *gene (ssr3383) encodes a short phycobilisome LC linker polypeptide and is the ultimate gene of a three-gene operon for phycobiliproteins. Cotranscription between this operon and SyR2 cannot be excluded unambiguously. However, a SyR2 transcript start was mapped within *apcC*, 49 nt before the end of the reading frame. This fact is less exotic than it seems. At the expected spacing six nt upstream, the transcript start is preceded by a regular TATA element (CAAAAT). Moreover, several examples indicate the location of ncRNA promoters within the protein-coding part of a gene: Transcription of the *ssrS *gene for 6S RNA in *E. coli *is initiated at two promoters, from these the distally located promoter P2 responds to σ70 and σS RNA polymerase holoenzymes and is located within the *ygfE *reading frame [35,36]. An example from *Synechocystis *is provided with IsrR, the antisense RNA that is initiated from within the gene *isiA*, although from the reverse complementary strand [18].

In a more general sense these results demonstrate that, just judging from the prediction, both candidate ncRNA genes might have been expected to be 3'UTRs due to their close location to an mRNA 3'end. We did not investigate their origin from a specific promoter further as we did for the Yfr2a ncRNA (see below), but the results shown in Fig. 4, in particular the lack of a longer transcript signal in the respective agarose gel blots, plus specific RACE signals confirm unambiguously that they do accumulate as individual small transcripts and therefore constitute *bona fide *ncRNAs.

### A family of ncRNAs that is widely conserved among cyanobacteria

**Figure 5:**
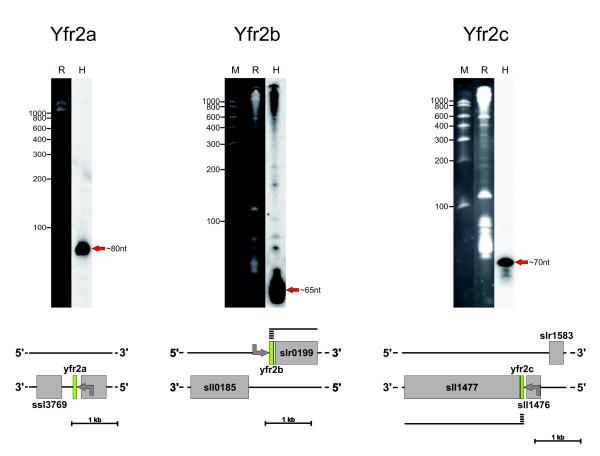
**All three predicted members of the Yfr2 family of ncRNAs are expressed in *Synechocystis *6803**. The three predicted ncRNAs belonging to cluster 219 are expressed in *Synechocystis *6803. The ncRNA Yfr2a is ~80 nt long. All initiation sites of transcription were mapped by 5' RACE (grey arrows). The transcriptional start site of Yfr2a was mapped unambiguously to position 1558975 (complementary strand), 93 nt downstream the end of the *trxA *(sll1980) open reading frame. Yfr2b and Yfr2c are initiated at positions 2730523 and 3398352 (complementary strand), respectively. All three ncRNAs are very strongly expressed in *Synechocystis *6803. However, the fact that it was not possible to map a specific initiation site of transcription for the genes slr0199 and sll1477 indicates their occasional or standard cotranscription with Yfr2b and Yfr2c. This assumption is further supported by some RNA fragments of higher molecular weight hybridizing with the Yfr2b probe. All labels are as in Fig. 4.

The vast majority of the ~100 bacterial ncRNAs experimentally verified thus far have been identified in *Escherichia coli *[2] and a few other model proteobacteria and *Pseudomonas *species. Therefore it is not surprising that, with the exception of the four highly conserved ncRNAs 6S RNA, tmRNA, ffs and RnpB, ortholog genes for ncRNAs are known only among very closely related species such as between *Salmonella *sp. and *Yersinia *sp. [37]. Here, with cluster 219 eight sequences were identified with high sequence and predicted secondary structure similarity to a family of ncRNAs initially found in marine *Prochlorococcus *[8]. There are four such ncRNAs in *Prochlorococcus *MED4 which in the original publication had been named Yfr2, Yfr3, Yfr4 and Yfr5 [8]. From the eight new members to this family in cluster 219 three belong to *Synechocystis*, one to *Thermosynechococcus *and two each are predicted in *Synechococcus *and in *Microcystis*. Since none of them has a more pronounced similarity to any of the original Yfr2-Yfr5 ncRNAs from *Prochlorococcus *MED4, we decided to call them all "Yfr2" according to the first member in this group and then just to add a suffix. Therefore, the three predicted candidates belonging to this ncRNA family in *Synechocystis *are Yfr2a, Yfr2b and Yfr2c. All three are expressed in *Synechocystis *(Fig. 5). The *yfr2a *gene is located downstream of thioredoxin A (*trxA*) gene sll1980. Both genes are in the same orientation but Yfr2a originates from a specific initiation site of transcription, mapped by TAP-RACE to position 1558975 in the genome (complementary strand), 94 nt 3' of the sll1980 stop codon (Fig. 5). The other two ncRNAs belonging to this family in *Synechocystis*, Yfr2b and Yfr2c, originate from genes directly upstream of two protein-coding genes, slr0199 and sll1477. In these two cases we mapped identical initiation sites of transcription for the ncRNAs and their respective downstream located protein-coding gene, to genomic positions 2730523 (forward strand, Yfr2b) and 3398352 (complementary strand, Yfr2c). Therefore, transcription of these two mRNAs occurs possibly by a read-through mechanism from Yfr2b or Yfr2c. Whether this type of transcriptional fusion has functional relevance is currently unknown as are the functions of slr0199 and sll1477. These genomic arrangements are not conserved as the genes adjacent to the other five candidate ncRNA genes belonging to this family differ in the other three cyanobacteria. This lack of synteny supports a function of Yfr2b and Yfr2c independent from serving merely as 5' untranslated leaders of slr0199 and sll1477.

**Figure 6:**
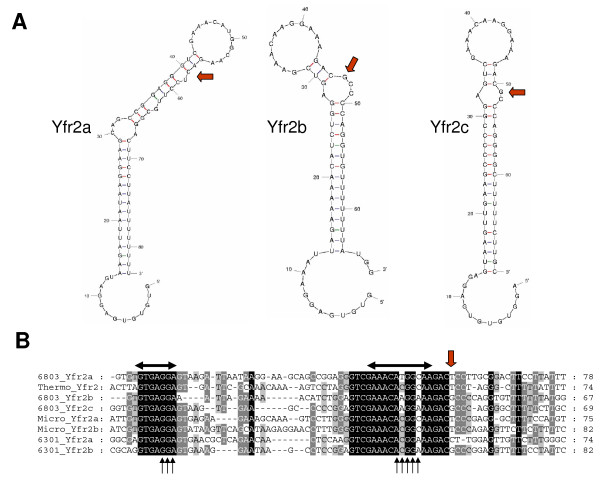
**Sequence alignments and secondary structure predictions of the 8 Yfr2-type ncRNAs identify conserved structure and sequence motifs**. **A**. Secondary structure predictions of the three experimentally confirmed ncRNAs Yfr2a, Yfr2b and Yfr2c from *Synechocystis *6803. They share a 12 nt central loop on a long helical stem that is interrupted by at least one bulge at position -4 with regard to this loop (red arrows). Moreover, the first 8–13 nt are predicted to be single-stranded. **B**. Alignments of all eight predicted Yfr2-type DNA sequences reveal two extremely conserved nucleotide stretches: the short unpaired region at the 5' end as well as the predicted centrally located loop element (labelled by horizontal black arrows). In contrast, the region between these two elements is not conserved in sequence or in its length. The single nucleotide breaking the stem at position -4 with regard to the loop is indicated by a red arrow. Note that the 3' end of the transcribed region has not been mapped. Those sequence stretches resembling "GGA" and "ANGGA" motifs are labelled by a set of black arrows. The non-*Synechocystis *6803 sequences are one from *Thermosynechococcus *(Thermo_Yfr2), two from *Microcystis *(Micro_Yfr2a and Micro_Yfr2b) and two from *Synechococcus *6301 (6301_Yfr2a and 6301_Yfr2b).

Sequence alignments and secondary structure predictions of the 8 Yfr2-5-type ncRNAs suggest a centrally located single-stranded loop element together with a short unpaired region at the 5' end that are highly conserved (Fig. 6). The long helical stem bearing the 12 nt loop is very characteristically predicted in all sequences to be interrupted by at least one bulge at position -4 with regard to this loop (Fig. 6). Interestingly, this feature is shared with the Yfr2-Yfr5 ncRNAs from marine cyanobacteria [8]. Bulge motifs have been recognized in a wide range of RNAs as key structural elements determining molecular recognition by other molecules [38]. Therefore, the conserved bulges in Yfr2-type ncRNAs may indicate their interaction with proteins. Indeed, another hint comes from the unpaired regions of these ncRNAs which resemble the extended "GGA" and "ANGGA" RsmA-binding motifs. The ncRNAs RsmX, RsmY and RsmZ found in *Pseudomonas *species contain several GGA and extended ANGGA motifs [39]. For RsmY, these motifs have been shown to be essential for sequestration of RsmA and its homolog RsmE in *Pseudomonas fluorescens *[40]. Non-coding RNAs containing this motif frequently have a titrating role on their target protein, regulating gene expression at the translational level. It was not possible, however, to identify RsmA and RsmE homologs in cyanobacteria.

### Expression analysis of Yfr2a

Starting with the mapped initiation site of Yfr2a we chose the region located immediately upstream of it in a promoter fusion experiment with *luxAB *genes to prove that it actually does contain a functional promoter. Moreover, if the expression of an ncRNA is regulated under certain environmental conditions this sometimes gives a hint into which processes this ncRNA might be involved in. As controls, we chose the same DNA fragment in reverse orientation and amplified and cloned the *psbA2 *(slr1311) promoter, again in both orientations.

**Figure 7:**
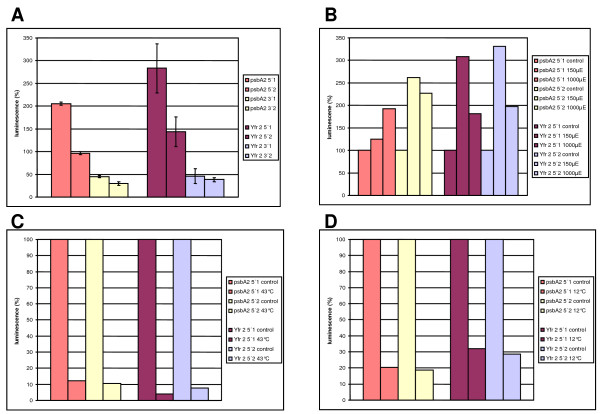
**Activity of the *Synechocystis *Yfr2a promoter under various conditions**. **A**. Fluorescence emitted from cultures with luciferase expression driven by the Yfr2a or the *psbA2 *(slr1311) promoter under standard growth conditions. All promoter fragments were used in the correct (5') or inverted (3') orientation with two independent clones each. Average absolute luminescence values together with standard deviations from three replicate measurements for each clone are shown. **B**. Measurement of luciferase activity 20 min after application of two different light treatments, transfer of cultures to 150 and 1,000 μmol photons s^-1 ^m^-2 ^for 15 minutes. **C**. Heat stress; expression was measured 20 min after exposure to 43°C for 10 min. **D**. Cold temperature stress; expression was measured 20 min after exposure to 12°C for 10 min. All measurements were performed with two independent clones in three replicates each. In B, C and D the values from the untreated controls were set to 100%. All cultures grew in late logarithmic phase during the measurements.

The 300 nt upstream of Yfr2a provided indeed very strong expression to the reporter genes – under all tested conditions the measured fluorescence values were comparable to those obtained from the *psbA2 *promoter-driven luciferase gene expression, whereas the reverse orientation of the same fragments provided very low activity only (Fig 7A). Under cold temperature (12°C) and heat (43°C) stress the activity of both promoters drops (Fig 7C, D), a possible pleiotropic effect. Under light stress, however, the activity of both promoters is stimulated. Whereas the activity of the *psbA2 *promoter is increased up to 250%, the Yfr2a promoter becomes activated up to 300% (Fig 7B). This is all the more striking since *psbA2 *frequently serves as an example for a typical light-inducible promoter.

### Six Clusters containing repetitive sequences

One problem when dealing with genome sequences of some cyanobacteria is the high number of repetitive sequences, mobile genetic elements and transposon-related sequences.

Indeed, the output from our prediction pipeline was contaminated by imperfect inverted repeat sequences flanking different families of IS elements, mainly in *Microcystis*, and to a lesser extent in *Synechocystis*. If subtracted from the data set, the total number of predicted RNA elements (numbers in brackets correspond to high-scoring elements) in *Synechocystis*, *Synechococcus*, *Thermosynechococcus *and *Microcystis *drops to 339 (78), 160 (53), 144 (42) and 426 (168). Such repetitive sequences were collected in six sequence clusters, namely #12, #25, #38, #68, #270 and #383 with 78, 87, 153, 83, 42 and 16 individual sequences, respectively. All 459 sequences from these clusters have been collected in a separate file and are accessible from our website [41].

## Conclusion

Comparative genomics-based prediction of ncRNA genes and candidate ncRNA genes is more and more becoming a standard tool to search for such genes within bacterial genomes [8,19-23].

Here we provide the first list of ncRNA and other RNA element candidates for model unicellular cyanobacteria. Surprisingly, we identified with Yfr2a-Yfr2c a family of ncRNAs which is widely conserved among cyanobacteria and which become accumulated to high concentrations.

Our experimental verification together with existing positive controls suggests a high number of positives in this candidate set. However, there are also putative 5' operon leaders, Rho-independent 3' transcriptional terminators and possibly yet unidentified riboswitches in this data set. Moreover, the output is contaminated to some extent by transposase-related sequences.

Nevertheless, by analogy to other bacteria, including the most streamlined marine cyanobacterium *Prochlorococcus *MED4 [9], this number of ncRNAs and other RNA elements is probably a grave underestimation. Therefore this analysis should be considered as a first step to become complemented by more exhaustive experimental screens, for instance by using tiling arrays or deep sequencing in the near future.

## Methods

### Cultures and manipulation of cyanobacteria

The *Synechocystis *Moscow strain was used in this study (obtained from A. Wilde, University of Giessen, Germany, originally from S. Shestakov, Moscow State University, Russia) and propagated on BG11 [42] 1% (w/v) agar (Bacto agar, Difco) plates. Liquid cultures of *Synechocystis *were grown at 30°C in BG11 medium under continuous illumination with white light of 50 μmol of photons•m-2•s-1 and a continuous stream of air.

As a promoter test vector we used the pILA plasmid [43] into which ~300 bp long promoter fragments were cloned as transcriptional fusions with the *luxAB *genes. After transformation, this plasmid integrates into the slr0168 gene within the chromosome of *Synechocystis *by homologous recombination. Transformation and analysis of correct integration and segregation was carried out as described elsewhere [44].

### Extraction and analysis of RNA

Exponentially growing *Synechocystis *cultures (OD750 0,6 – 0,8) were collected by filtration (Pall Supor 800 Filter). Filters with cells were dissolved in 1 ml Trizol per 40 ml culture, immediately frozen in liquid nitrogen and incubated for 15 min at 65°C in a water bath. Further RNA isolation followed the manufacturer's protocol.

Small RNA Northern blots were prepared from the separation of 10 to 25 μg of total RNA on 10% urea-polyacrylamide gels as described by Steglich *et al. *[9]. Polyacrylamide gels were stained with ethidium bromide (0.3 μg/l) in 1× TBE buffer, rinsed with 1× TBE and analyzed with an E-BOX video gel documentation system (Peqlab). Transcript sizes were determined by correlation to Fermentas' RiboRuler low range RNA marker. Blots for RNAs with higher molecular weight were prepared from the separation of 5 μg of total RNA on 1,5% denaturing agarose gels. Transcriptional start sites were determined by 5'-RACE as described [9].

### Sequence data

Genome sequences were obtained from the finished microbial genomes website at Genbank [45] with the following accession numbers: *Synechococcus elongatus *PCC 6301, NC_006576; *Synechocystis *sp. PCC 6803, NC_000911; *Thermosynechococcus elongatus *BP-1, NC_004113; *Microcystis aeruginosa *NIES843, NC_010296.

### Prediction of RNA elements in a comparative approach

We performed a comparative prediction of ncRNA elements within intergenic regions (IGRs). Therefore, all IGRs longer than 50 nt were extracted and compared among the different genomes using Blast. Intragenomic analyses with the settings given in Fig. 1 revealed a high number of repetitive sequences in some of the analyzed genomes. There were 895 intragenomic hits within the genome of *Synechococcus*, 2227 hits within *Thermosynechococcus*, 7198 in *Synechocystis *and 557014 mutual hits of IGRs from *Microcystis *with other IGRs from the same organism. A large number of repetitive elements N would produce approximately N^2 ^hits, meaning that the square root of the number of hits gives an estimate of the number of repetitive elements. In the case of *Microcystis *this yields ~746 repetitive elements, which is supported by the finding of six large clusters holding 383 sequences from *Microcystis*. Therefore, we refrained from searching for intragenomic similarity. Based on these results homologous sequence regions got clustered together, aligned using ClustalW and analyzed for structural significance by RNAz. Alignments were postprocessed using the tool rnazSelectSeqs.pl with default parameters from the RNAz package. RNAz was applied in a sliding window approach (a step size of 10 nt and different window sizes were used, namely 80,100,120,140 and 160 nt) of which the window with highest probability was selected. Elements are termed "High-scoring" if they achieve an RNAz probability of 0.5 or more or if their Z-score is -2.0 or below. Details about the individual steps and their outcome are shown in Fig. 1. All predictions can be found at [25] and [41].

### Matching predictions to Rfam and TransTermHP

All individual sequences were matched against Rfam [27] using the batch search feature provided by Rfam. Mapping of predicted sequences to information about Rho-independent terminators provided by TransTermHP [28] was done using Vmatch [46]. Therefore, TransTermHP predictions for *Microcystis *were computed using TransTermHP 2.06 with default parameters, while for *Synechocystis*, *Synechococcus *and *Thermosynechococcus *existing predictions were downloaded from the TransTermHP website. All predictions were converted to FASTA-format and searched for at least 30 nt long hits with 100% identity to candidate sequences.

### Comparative sequence/structure analysis

Multiple sequence alignments were generated using ClustalW [47] with default parameters for DNA. Comparative structure prediction was done with RNAlishapes [48], a tool which predicts a consensus structure for a set of aligned sequences by taking covariance and free energy into account. The resulting consensus structure was analysed together with the multiple sequence alignment using RALEE [49]. The latter served also for manual optimisation of the alignment and the consensus structure, respectively, and for the production of colour annotated alignments. Colour plots of Consensus structures were generated using RNAalifold [50].

### Oligonucleotides

Oligonucleotide primers for the generation of hybridization probes (T7 promoter sequence in boldface letters):

Yfr2c_for: 5'-**TAATACGACTCACTATAGG**ccgccagcgccattgcttc-3'

Yfr2c_rev: 5'-cttaggacaggtgtgaggaaattag-3'

Yfr2b_for: 5'-**TAATACGACTCACTATAGG**cggggagcatagaccagcttg-3'

Yfr2b_rev: 5'-ggaagttattatctagaggtgtgtgag-3'

Yfr2a_for: 5'-**TAATACGACTCACTATAGG**aggcaaaaaaataaggaagtccgcaag-3'

Yfr2a_rev: 5'-cggctatcccgcccttagg-3'

Syr1_for: 5'-**TAATACGACTCACTATAGG**ccgagggcatatctaggagaac-3'

Syr1_rev: 5'-ggctatggaaacccgacagaattc-3'

Syr2_for: 5'-**TAATACGACTCACTATAGG**caaacaaaaaaagaggccattgctgacc-3'

Syr2_rev: 5'-gactagttgttgctaatttagcaatgttg-3'

Oligonucleotides for promoter fusion experiments (*Kpn*I restriction sites 5'-GGTACC-3' introduced for cloning are labelled in boldface letters):

Yfr2a (fw): 5'-**GGTACC**CTAGATGACACCGGCACG-3'

Yfr2a (rev): 5'-**GGTACC**CTCCTCACACACAAATAAATGTTAG-3'

psbA2 (fw): 5'-CCTT**GGTACC**AAGAGTAATGGCGTGC-3'

psbA2 (rev): 5'-GATT**GGTACC**GGAACTGACTAAACTTAGTC-3'

Oligonucleotides for specific mapping of 5'ends through 5'RACE:

Yfr2c_5'RT: 5'-CCTAAAAATTGCCATAAAAAAACAC-3'

Yfr2c_5'Race: 5'-TCTTTCCTTGTTTCGACTCCAG-3'

sll1477_5'RT: 5'-GCGGCCAGAGGTTTCC-3'

sll1477_5'Race: 5'-CAGCGTAGCTAGGGAAATCACCACCAG-3'

Yfr2b_5'RT: 5'-AAAAGGCAAGAAAAAGCCCC-3'

Yfr2b_5'Race: 5'-TTTCCTTGTTTCGACTCCGGGG-3'

slr0199_5'RT: 5'-TGACCCAGATACCCTAAAAG-3'

slr0199_5'Race: 5'-CTTTTGATAATCTTGGCGGCC-3'

Yfr2a_5'RT: 5'-GGAGTCTTGCCATGTTTCG-3'

Yfr2a_5'Race: 5'-CCTCCGGCTGCTTCCTT-3'

### Hybridization conditions

Northern hybridization was performed at 62°C in hybridization buffer (50% deionized formamide, 7% SDS, 250 mM NaCl, 120 mM Na(PO_4_), pH 7.2) as described by Steglich *et al. *[9]. Single stranded probes were generated from PCR-amplified templates incorporating the T7 promoter in one of the oligonucleotide primers, using the MAXIscript Kit (Ambion, USA) and 100 ng PCR-generated DNA template.

Detailed information on all clusters predicted by comparative genome analysis including the positions of all sequences can be found at: [25] and [41].

## Abbreviations

IGR: intergenic region; ncRNA: non-coding RNA.

## Authors' contributions

BV designed and carried out bioinformatic analyses and participated in drafting the manuscript, JG performed all RNA analyses in the laboratory, SU and VS constructed the promoter test constructs and performed the reporter gene assays. WRH designed research and wrote the manuscript. All authors read and approved the final manuscript.
